# Supplementary material for: Structural basis for the H2AK119ub1-specific DNMT3A-nucleosome interaction
Source: Nat Commun. 2024 Jul 23;15:6217. doi: 10.1038/s41467-024-50526-3 (PMC11266573; doi:10.1038/s41467-024-50526-3)
Supplement: Supplementary file 1 — Supplementary Information [file 41467_2024_50526_MOESM1_ESM.pdf]

Supplementary Information

Structural basis for the H2AK119ub1-specific DNMT3A-nucleosome interaction

Xinyi Chen[1,7], Yiran Guo[2,3,7], Ting Zhao[4], Jiuwei Lu[1], Jian Fang[1], Yinsheng Wang[4,5], Gang Greg Wang[2,3,6,*], Jikui Song[1,*]

[1]Department of Biochemistry, University of California, Riverside, CA 92521, USA

[2]Department of Pharmacology and Cancer Biology, Duke University School of Medicine, Durham, NC 27710, USA

[3]Duke Cancer Institute, Duke University School of Medicine, Durham, NC 27710, USA

[4]Environmental Toxicology Graduate Program, University of California, Riverside, CA 92521, USA

[5]Department of Chemistry, University of California, Riverside, CA 92521, USA

[6]Department of Pathology, Duke University School of Medicine, Durham, NC 27710, USA

[7]These authors contributed equally to this work

*Correspondence: greg.wang@duke.edu; jikui.song@ucr.edu

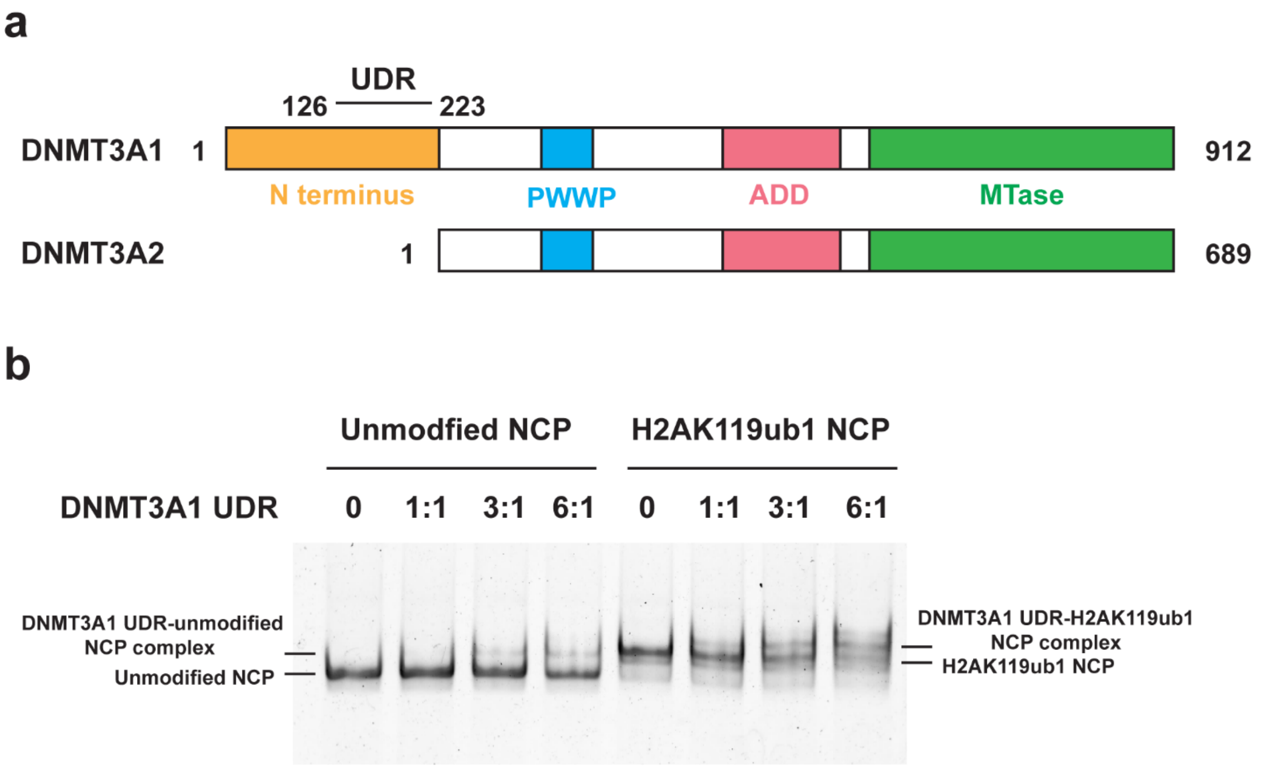

Supplementary Figure 1. Biochemical analysis of the H2AK119ub1-dependent binding between DNMT3A1 UDR and nucleosome. (**a**) Domain architecture of DNMT3A1 and DNMT3A2, with individual domains color coded. The H2AK119ub1-binding DNMT3A1 UDR, used for structural and biochemical characterizations in this study, is delimited by residue numbers. (**b**) EMSA analysis assessing the binding of wild-type (WT) DNMT3A1 UDR to either unmodified (left) or H2AK119ub1-modified (right) NCP. The samples were prepared in 1:1, 3:1 and 6:1 molar ratio of DNMT3A1 UDR:NCP. NCP alone was loaded as a control.

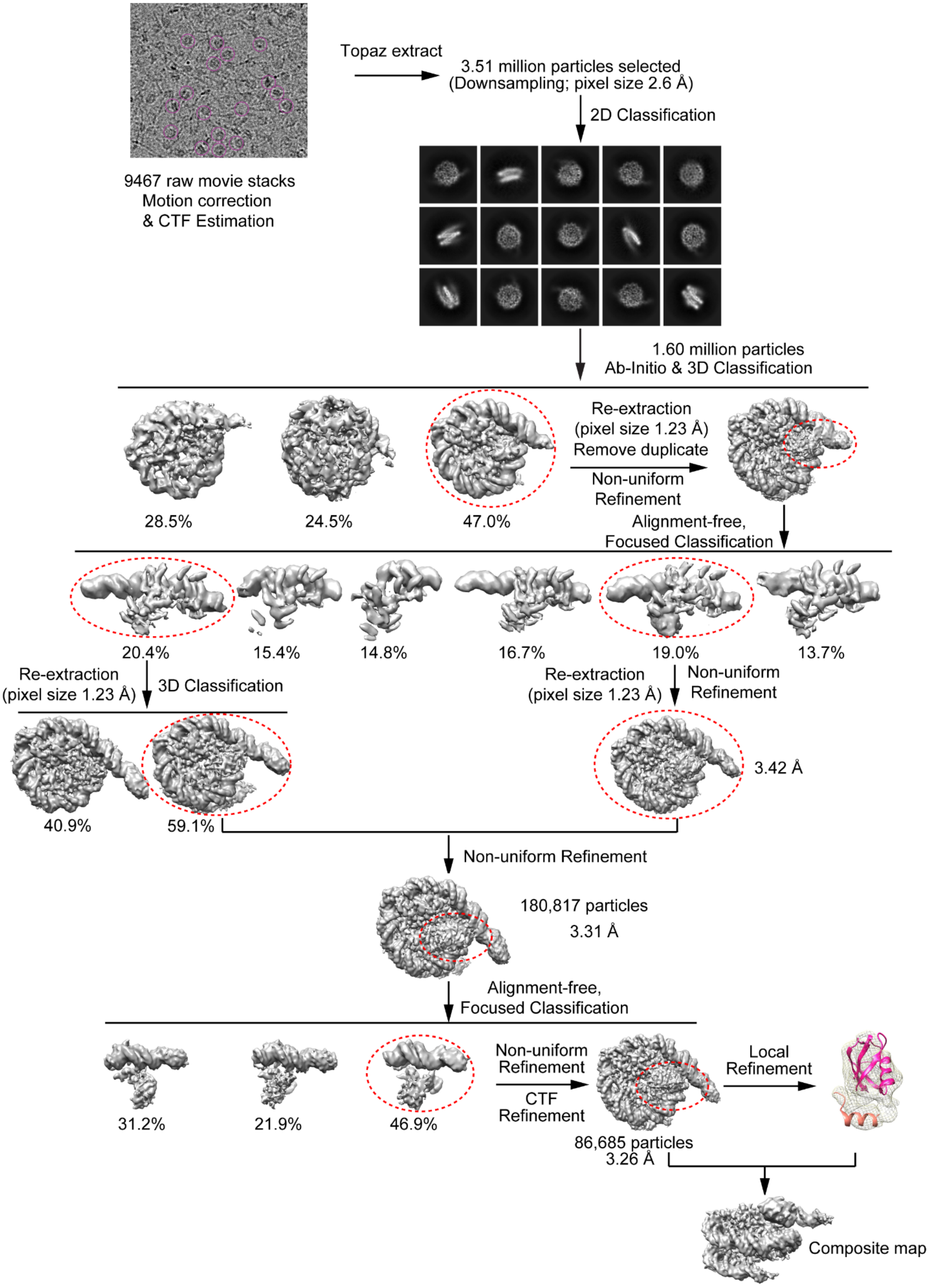

Topaz extract
3.51 million particles selected
(Downsampling; pixel size 2.6 Å)
2D Classification
9467 raw movie stacks
Motion correction
& CTF Estimation
1.60 million particles
Ab-Initio & 3D Classification
Re-extraction
(pixel size 1.23 Å)
Remove duplicate
Non-uniform
Refinement
28.5%
24.5%
47.0%
Alignment-free,
Focused Classification
20.4%
15.4%
14.8%
16.7%
19.0%
13.7%
Re-extraction
(pixel size 1.23 Å)
3D Classification
Re-extraction
(pixel size 1.23 Å)
Non-uniform
Refinement
3.42 Å
40.9%
59.1%
Non-uniform Refinement
180,817 particles
3.31 Å
Alignment-free,
Focused Classification
31.2%
21.9%
46.9%
Non-uniform
Refinement
CTF
Refinement
Local
Refinement
86,685 particles
3.26 Å
Composite map

Supplementary Figure 2. Cryo-EM data processing workflow for DNMT3A1 UDR in complex with H2AK119ub1-modified NCP.

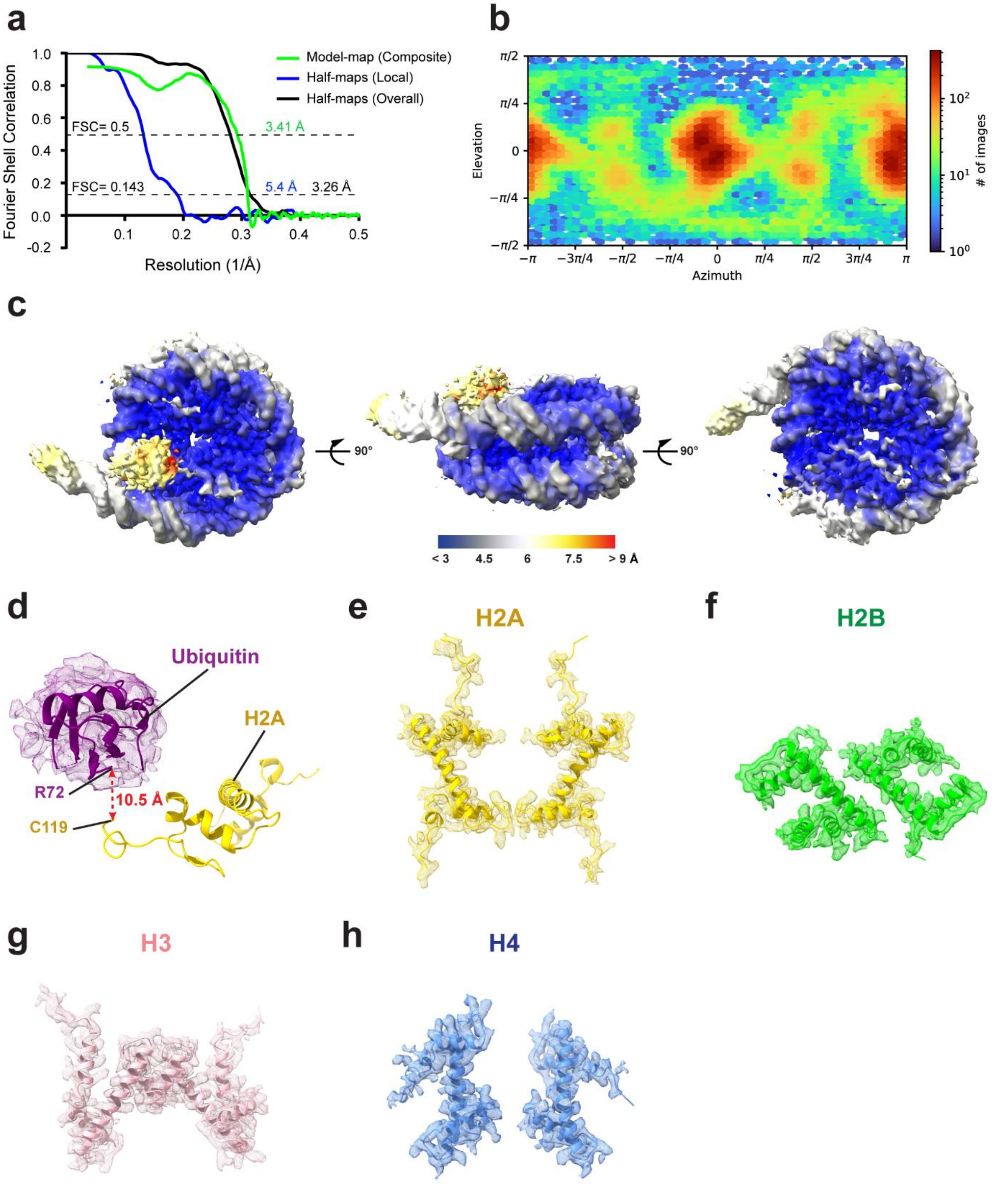

Supplementary Figure 3. Cryo-EM reconstruction of the complex between DNMT3A1 UDR and H2AK119ub1-modified NCP. (a) Fourier shell correlation (FSC) curves of overall, local and composite maps for the DNMT3A1 UDR–H2AK119ub1-modified NCP complex as a function of resolution, with the map resolution at FSC=0.143

(for overall and local maps) and model-map resolution at FSC=0.5 for composite map indicated. (**b**) Orientation distribution map for the DNMT3A1 UDR–H2AK119ub1-modified NCP complex. (**c**) Local resolution map for the DNMT3A1 UDR–H2AK119ub1-modified NCP complex. (**d**) Density map and atomic model for H2AK119ub1. The H2AK119C-conjugated ubiquitin is positioned such that the C-terminal residue R72 of ubiquitin is in ~10.5-Å distance to H2AK119C. (**e-h**) Density map and atomic model for H2A (e), H2B (f), H3 (g) and H4 (h).

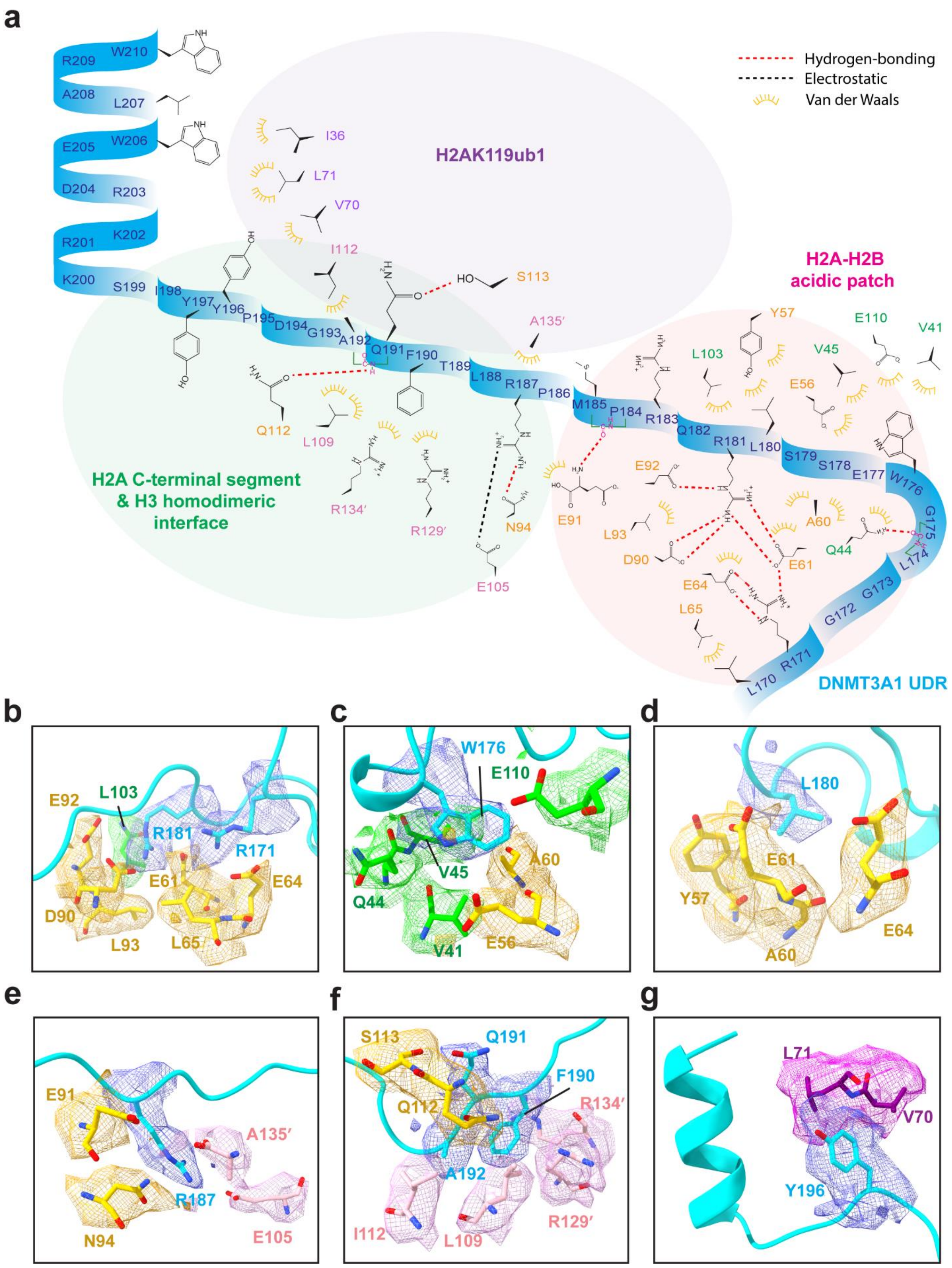
a
b
c
d
e
f
g
Hydrogen-bonding
Electrostatic
Van der Waals
H2AK119ub1
H2A-H2B
acidic patch
H2A C-terminal segment
& H3 homodimeric
interface
DNMT3A1 UDR
W176
E110
L103
E92
R181
R171
E61
E64
D90
L93
L65
V45
A60
Q44
V41
E56
L180
Y57
E91
A135′
R187
N94
E105
S113
Q191
F190
Q112
R134′
A192
I112
L109
R129′
L71
V70
Y196

Supplementary Figure 4. Structural details of the interaction between DNMT3A1 UDR and H2AK119ub1-modified NCP. (**a**) Schematic view of DNMT3A1 UDR–H2AK119ub NCP interaction. Hydrogen-bonding and electrostatic interactions are indicated by red and black dashed lines, respectively. Van der Waals contacts are shown as orange gears. (**b-g**) Atomic model and density map for the interaction between DNMT3A1 UDR and H2AK119ub1-modified NCP involving DNMT3A1 R171 and R181 (b), W176 (c), L180 (d), L187 (e), F190, Q191 and A192 (f), and Y196 and W206 (g).

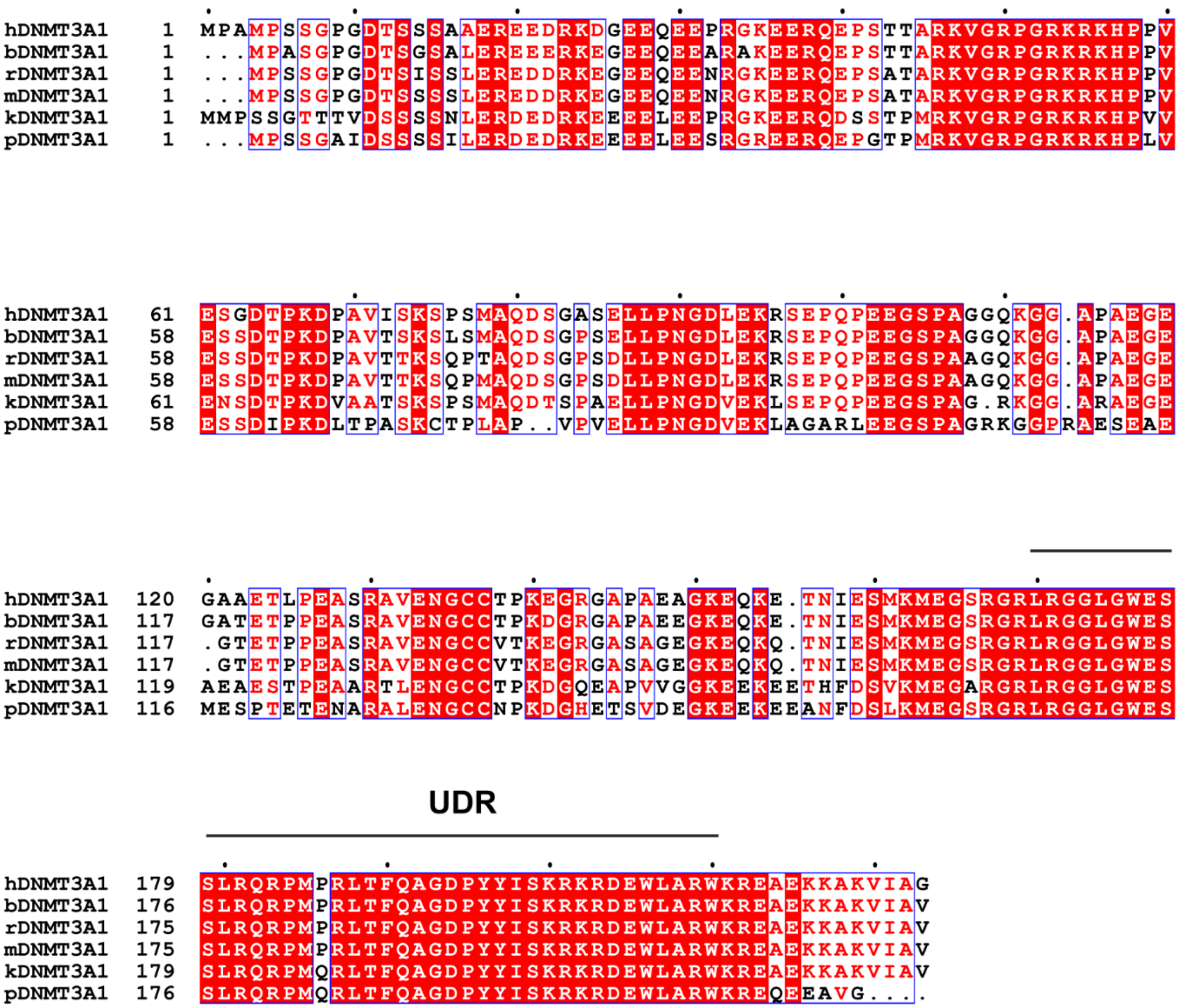

Supplementary Figure 5. Sequence alignment of the N-terminal domain of DNMT3A1 from various species. The aligned sequences include the N-terminal domain of DNMT3A1 from human (hDNMT3A1), bovine (bDNMT3A1), rat (rDNMT3A1), mouse (mDNMT3A1), koala (kDNMT3A1) and platypus (pDNMT3A1). Identical residues are colored white in red background and similar residues are colored red. The DNMT3A1 UDR region was indicated on top of the sequences.

a

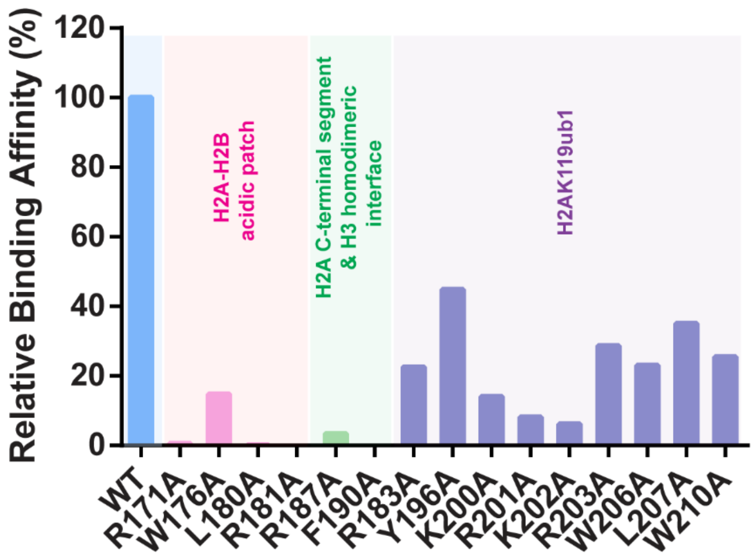

b

Mutant Variant	K_d	Fold Change of K_d
WT	16.6 ± 2.2 nM	1
**Interaction with H2A-H2B acidic patch**		
R171A	3.2 ± 0.5 μM	193
W176A	113 ± 43 nM	6.8
L180A	11.4 ± 3.8 μM	687
R181A	N.M.	N.M.
**Interaction with H2A C-terminal segment and H3 homodimeric interface**		
R187A	498 ± 190 nM	30.0
F190A	25.0 ± 5.2 μM	1506
**Interaction with nucleosomal DNA and H2AK119ub1**		
R183A	73.1 ± 25.9 nM	4.4
Y196A	37.1 ± 20.1 nM	2.2
K200A	118.0 ± 74.1 nM	7.1
R201A	204 ± 24 nM	12.3
K202A	272 ± 42 nM	16.4
R203A	57.9 ± 11.0 nM	3.5
W206A	72.0 ± 22.1 nM	4.3
L207A	47.3 ± 3.1 nM	2.8
W210A	65.3 ± 2.0 nM	3.9

Supplementary Figure 6. Summary of BLI binding assays for the DNMT3A1 UDR–H2AK119ub1 NCP interaction. (**a**) Relative binding affinities of DNMT3A1 UDR, WT or mutant, to H2AK119ub1-modified NCP, with mutations grouped based on the corresponding H2AK119ub1 NCP-binding regions. (**b**) Summary of dissociation constant (K_d) of DNMT3A1 UDR, WT or mutant, with H2AK119ub1 NCP, as well as the fold change of the K_d values relative to that of WT DNMT3A1 UDR, measured by BLI binding assays.

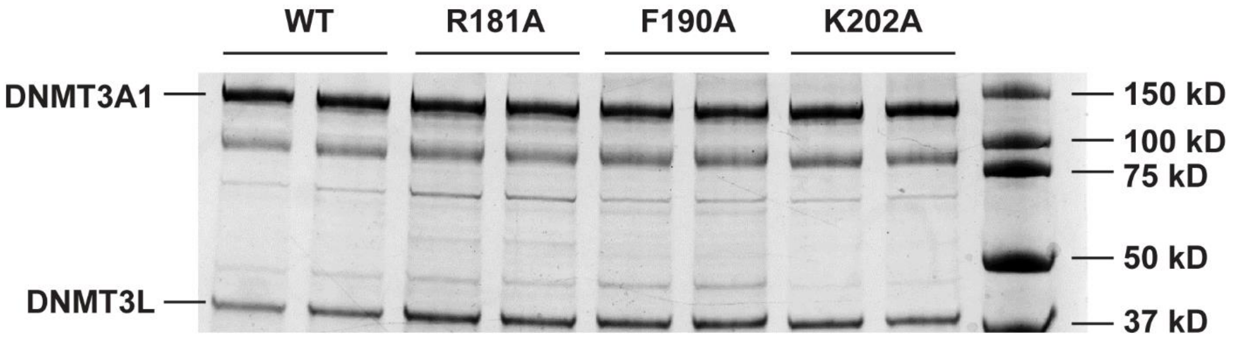

Supplementary Figure 7. SDS-PAGE images of full-length DNMT3A1, WT or mutant. The bands for full-length DNMT3A1 and DNMT3L are indicated on the left. The molecular weight markers are labeled on the right.

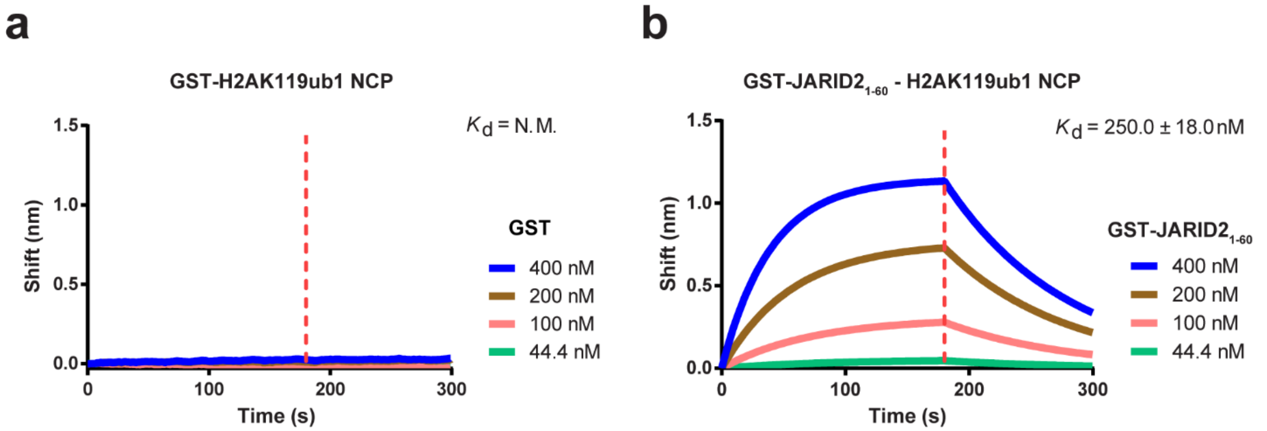

Supplementary Figure 8. BLI analysis of the binding between GST-tagged JARID2$_{1-60}$ and H2AK119ub1-modified NCP. (**a**,**b**) BLI binding assay for GST-tag (a) or GST-JARID2$_{1-60}$ (b) with H2AK119ub1-modified NCP. The concentrations of GST or GST-JARID2$_{1-60}$ used in the assays are indicated on the right. N.M., not measurable. Data are mean ± s.d. (n = 2 biological replicates). One representative set of data is shown.

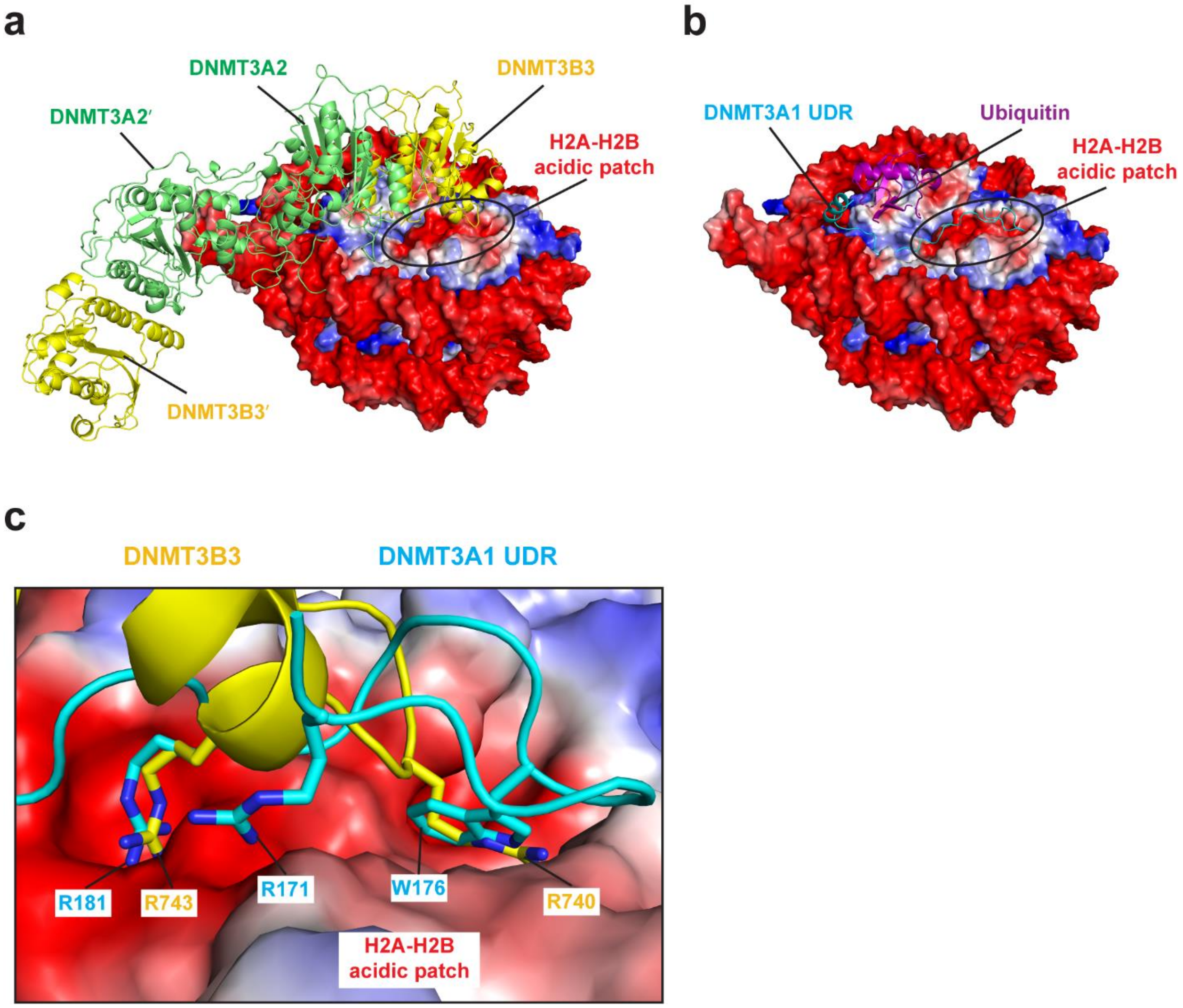

Supplementary Figure 9. Structural comparison of the DNMT3A2–DNMT3B3–NCP and DNMT3A1 UDR–H2AK119ub1-modified NCP complexes. (**a**) Electrostatic surface of NCP bound to DNMT3A2-DNMT3B3 (PDB 6PA7). (**b**) Electrostatic surface of H2AK119ub1-modified NCP bound to DNMT3A1 UDR. (**c**) Close-up view of the structural overlay of DNMT3A1 UDR–H2AK119ub1 NCP and DNMT3A2–DNMT3B3–NCP complexes highlighting the competitive binding of DNMT3A1 UDR and DNMT3B3 to the H2A-H2B acidic patch. For clarity, the structure of DNMT3A2–DNMT3B3-bound NCP is not shown. The H2A-H2B acidic patch-interacting residues are shown in stick representation.

For Supplementary Figure 1b

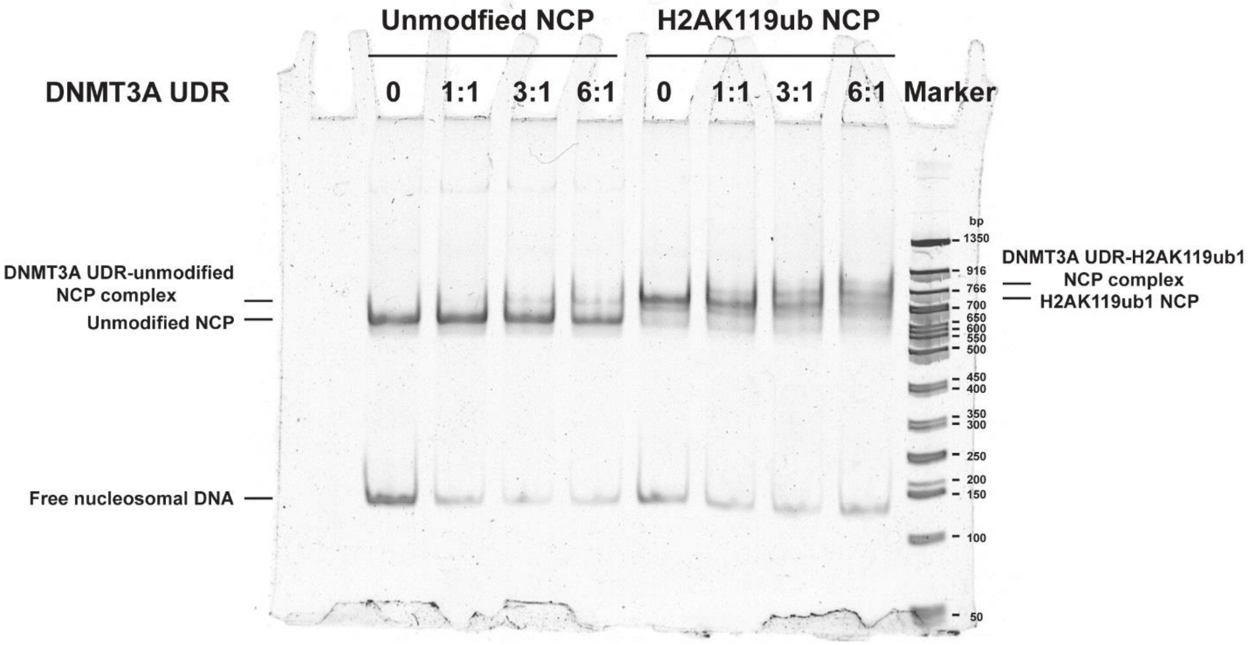

For Supplementary Figure 7

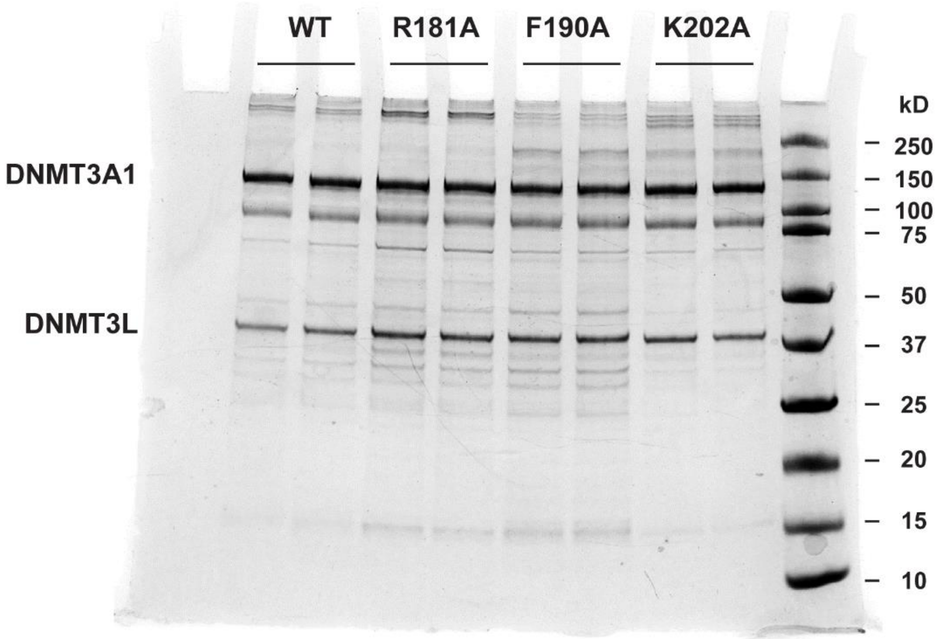

Supplementary Figure 10. Raw gel images used in this study.

Supplementary Table 1. Cryo-EM data collection, structure refinement and validation statistics

Codes	EMD-41920 (Consensus), EMD-41921 (Local), EMD-41922 (Composite), 8U5H (pdb)
**Data collection and processing**	
Microscope	Titan Krios
Camera	TFS Falcon IV
Magnification	130,000
Voltage (kV)	300
Defocus range (μm)	-0.8 ~ -2.5
Exposure time (s)	7
Dose rate (e^{-}/Å^{2}/s)	7
Number of frames	40
Pixel size (Å)	0.926
Micrographs (no.)	13,419
Initial particles (no.)	1,797,273
Symmetry imposed	*C1*
Final particles (no.)	86,685
Map resolution (Å)	3.26 (Consensus), 5.4 (Local)
FSC threshold	0.143
**Refinement**	
Initial model used	6WKR
Model resolution (Å)	3.50
FSC threshold	0.5
Map sharpening *B* factor (Å^{2})	-30 (Consensus), -100 (Local), NA (Composite)
CC (mask)	0.83
Model composition	
Non-hydrogen atoms	13436
Protein residues	903
Nucleotides	314
B factors (Å^{2})	
Protein	62.87
Nucleotide	131.46
R.m.s. deviations	
Bond lengths (Å)	0.005
Bond angles (°)	0.710
**Validation**	
MolProbity score	1.95
Clash score	13.88
Rotamer outliers (%)	0.85
Ramachandran plot	
Favored (%)	95.44
Allowed (%)	4.56
Disallowed (%)	0.00
