# Supplementary material for: Structural basis for the H2AK119ub1-specific DNMT3A-nucleosome interaction
Source: Nat Commun. 2024 Jul 23;15:6217. doi: 10.1038/s41467-024-50526-3 (PMC11266573; doi:10.1038/s41467-024-50526-3)
Supplement: Supplementary file 3 — Description of Additional Supplementary Files [file 41467_2024_50526_MOESM3_ESM.pdf]

Description of Additional Supplementary Files

File Name: Supplementary Data 1
Description: Selected-ion chromatograms (SICs) for monitoring the neutral loss of a 2-deoxyribose from the $[M+H]^+$ ions of 5-mdC (m/z 242 → 126), [$^{13}C_5$]-5-mdC (m/z 247 → 126), dG (m/z 268 → 152) and [$^{15}N_5$]-dG (m/z 273 → 157) for EV (a), WT (b), R181A (c) and R190A (d), respectively.
