# Supplementary material for: Structural basis for the H2AK119ub1-specific DNMT3A-nucleosome interaction
Source: Nat Commun. 2024 Jul 23;15:6217. doi: 10.1038/s41467-024-50526-3 (PMC11266573; doi:10.1038/s41467-024-50526-3)
Supplement: Supplementary file 5 — Reporting Summary [file 41467_2024_50526_MOESM5_ESM.pdf]

nature research

Corresponding author(s): Gang, G. Wang, Jikui Song

Last updated by author(s): Jun 7, 2024

Reporting Summary

Nature Research wishes to improve the reproducibility of the work that we publish. This form provides structure for consistency and transparency in reporting. For further information on Nature Research policies, see Authors & Referees and the Editorial Policy Checklist .

Statistics

For all statistical analyses, confirm that the following items are present in the figure legend, table legend, main text, or Methods section.

n/a	Confirmed	
☐	☒	The exact sample size (*n*) for each experimental group/condition, given as a discrete number and unit of measurement
☐	☒	A statement on whether measurements were taken from distinct samples or whether the same sample was measured repeatedly
☐	☒	The statistical test(s) used AND whether they are one- or two-sided<br>*Only common tests should be described solely by name; describe more complex techniques in the Methods section.*
☒	☐	A description of all covariates tested
☒	☐	A description of any assumptions or corrections, such as tests of normality and adjustment for multiple comparisons
☐	☒	A full description of the statistical parameters including central tendency (e.g. means) or other basic estimates (e.g. regression coefficient) AND variation (e.g. standard deviation) or associated estimates of uncertainty (e.g. confidence intervals)
☐	☒	For null hypothesis testing, the test statistic (e.g. *F*, *t*, *r*) with confidence intervals, effect sizes, degrees of freedom and *P* value noted<br>*Give P values as exact values whenever suitable.*
☒	☐	For Bayesian analysis, information on the choice of priors and Markov chain Monte Carlo settings
☒	☐	For hierarchical and complex designs, identification of the appropriate level for tests and full reporting of outcomes
☐	☒	Estimates of effect sizes (e.g. Cohen's *d*, Pearson's *r*), indicating how they were calculated

Our web collection on statistics for biologists contains articles on many of the points above.

Software and code

Policy information about availability of computer code

Data collection	Cryo-EM data were collected using the Leginon software at Nation Center for CryoEM Access and Training (NCCAT).
Data analysis	For structural study, cryoSPARC v4.0.1 was used for image processing, particle classification, density refinement, local resolution estimation and map sharpening. Relion v4.1 was used for particle subtraction and alignment-free 3D classification. UCSF ChimeraX v1.5 and Coot v0.9.6 were used for modeling building. PHENIX v1.20.1 was used for Real-Space structure calculation, and Pymol v2.5.2 was used for structural analysis. GraphPad Prism v6.01 was used for graph plotting. GatorOne v2.10.4 was used for BLI data analysis.

For manuscripts utilizing custom algorithms or software that are central to the research but not yet described in published literature, software must be made available to editors/reviewers. We strongly encourage code deposition in a community repository (e.g. GitHub). See the Nature Research guidelines for submitting code & software for further information.

Data

Policy information about availability of data

All manuscripts must include a data availability statement. This statement should provide the following information, where applicable:

- Accession codes, unique identifiers, or web links for publicly available datasets
- A list of figures that have associated raw data
- A description of any restrictions on data availability

The consensus, local and composite 3D cryo-EM maps for the DNMT3A UDR-H2AK119ub1 NCP complex have been deposited in the Electron Microscopy Data Bank under the accession numbers EMD-41920, EMD-41921 and EMD41922, respectively. Atomic coordinates for the structural models have been deposited in the Protein Data Bank under accession code 8U5H.

Field-specific reporting

Please select the one below that is the best fit for your research. If you are not sure, read the appropriate sections before making your selection.

- [x] Life sciences
- [ ] Behavioural & social sciences
- [ ] Ecological, evolutionary & environmental sciences

For a reference copy of the document with all sections, see nature.com/documents/nr-reporting-summary-flat.pdf

Life sciences study design

All studies must disclose on these points even when the disclosure is negative.

Sample size	Biochemical assays were completed using wild type or mutants of DNMT3A fragments. The sample size is sufficient to delineate the mutational effects of DNMT3A.
Data exclusions	No data exclusion.
Replication	The BLI binding and chromatin fractionation assays were performed twice with consistent results. The DNA methylation assays were performed in triplicates with consistent results.
Randomization	The assays performed in this study require a rational approach for activity comparison. Therefore, randomization is not applicable to our experimental set up.
Blinding	The assays performed in this study require rational design. Therefore, blinding is not applicable to any biochemical or cellular assay performed in this study.

Reporting for specific materials, systems and methods

We require information from authors about some types of materials, experimental systems and methods used in many studies. Here, indicate whether each material, system or method listed is relevant to your study. If you are not sure if a list item applies to your research, read the appropriate section before selecting a response.

Materials & experimental systems

n/a	Involved in the study	
☐	☒	Antibodies
☐	☒	Eukaryotic cell lines
☒	☐	Palaeontology
☒	☐	Animals and other organisms
☒	☐	Human research participants
☒	☐	Clinical data

Methods

n/a	Involved in the study	
☒	☐	ChIP-seq
☒	☐	Flow cytometry
☒	☐	MRI-based neuroimaging

Antibodies

Antibodies used	Antibodies used for include GAPDH Antibody (Cell Signalling #2118L), H3 (Cell Signalling #4499S), DNMT3A (Abcam ab2850), and anti-GFP (Abcam, ab290), and anti-rabbit Alexa-488 conjugated secondary antibody (Invitrogen, #A-11008)
Validation	All antibodies used are commercially available and validated by corresponding companies.<br>GAPDH antibody: https://www.cellsignal.com/products/primary-antibodies/gapdh-14c10-rabbit-mab/2118<br>H3 antibody: https://www.cellsignal.com/products/primary-antibodies/histone-h3-d1h2-xp-rabbit-mab/4499<br>DNMT3A antibody: https://www.abcam.com/products/primary-antibodies/dnmt3a-antibody-ab2850.html<br>anti-GFP: https://www.abcam.com/products/primary-antibodies/gfp-antibody-ab290.html

Eukaryotic cell lines

Policy information about cell lines

Cell line source(s)	Mouse embryonic stem cells with triple knockout of Dnmt1, Dnmt3a and Dnmt3b, were cultivated.
Authentication	Authentication of cell line identity, including that of parental and derived lines, was ensured by Tissue Culture Facility affiliated to the Duke University School of Medicine using the genetic signature profiling and fingerprinting analysis.
Mycoplasma contamination	Every 1-2 month, a routine examination of cell lines in culture for any possible mycoplasma contamination was carried out using MycoAlert Mycoplasma Detection Kit (Lonza). No mycoplasma contamination was identified.

Commonly misidentified lines<br>(See ICLAC register)	No commonly misidentified cell lines were used in the study.
